# A Protean Insect Receptor Holds the Key to Broad-Based Pathogen Recognition

**DOI:** 10.1371/journal.pbio.0040246

**Published:** 2006-06-20

**Authors:** Liza Gross

Anything that's alive runs the risk of infection. How you respond to infection, however, depends on where you sit on the evolutionary tree. Humans and other vertebrates can fend off billions of pathogens by routinely recombining bits of genes for surface molecules on the cells charged with pathogen recognition. Insects and other invertebrates rely to a large degree on the pathogen recognition molecules (called pattern recognition receptors) they were born with. When a pattern recognition receptor detects a pathogen—based on what's known as its pathogen-associated molecular pattern—the receptor can launch a direct attack that either engulfs the invader, through encapsulation or phagocytosis, or triggers signaling pathways that regulate immune system genes involved in killing the pathogen.

In a new study, Yuemei Dong, Harry Taylor, and George Dimopoulos found a mosquito gene that vastly boosts the ability of insect pattern recognition receptors to detect pathogens. Originally implicated in neuron development, the gene can create a plethora of receptors for the malaria vector *Anopheles gambiae*. The *AgDscam* gene—short for *Anopheles gambiae* Down syndrome cell adhesion molecule gene—has 101 protein-coding regions (called exons) that can be mixed and matched after transcription to produce over 31,000 possible alternative splice forms with different properties. Thus, while B cell and T cell receptor diversity is generated largely at the gene sequence level before transcription, *AgDscam* diversity is produced by reshuffling sections of gene transcripts before translation into protein.

*Dscam* was first characterized in the fruitfly *(Drosophila melanogaster)*, where it can generate about 38,000 splice forms with different recognition and binding specificities from 95 variable exons. It's been suggested that a diverse inventory of adhesion molecules may help olfactory nerves establish the proper connections during development. But the presence of high levels of *Dscam* in cells that function in the fly's innate immune system and evidence of involvement in phagocytosis raised the possibility that the gene also plays a diverse recognition role in immunity.

Dong et al. found that *AgDscam*, like the fly version, has three variable regions within a portion of the immunoglobulin (Ig) gene. Each region contains different numbers of alternative splicing exons: Ig4 has 14, Ig6 has 30, and Ig10 has 38, leading to a possible 31,920 alternative splice forms. The researchers worked with a mosquito immune cell line to investigate *AgDscam*'s response to infection. Exposure to bacteria, fungi, and parasite surface molecules caused the cells to produce different *AgDscam* splice-form repertoires with different interaction properties. As with the cell lines, bacterial infection of adult mosquitoes also caused alternative splicing of *AgDscam*. Infecting mosquitoes with two different *Plasmodium* malaria parasites produced completely different *AgDscam* splice-form repertoires.

When the researchers cut *AgDscam* protein levels in half with a technique that “silences” a gene by degrading its transcript, they could link its function with phagocytosis of pathogens. Mosquitoes with a silenced *AgDscam* gene succumbed to bacterial infections (caused by two types of bacteria that produce different surface proteins) at much higher rates than did mosquitoes with a functioning *AgDscam* gene. Silencing *AgDscam* also resulted in a “profound proliferation” of opportunistic microbes, suggesting its essential role in defending the mosquito against bacterial infections. When gene-silenced mosquitoes fed on blood infected with malaria parasites, the researchers found a 65% increase of parasites on the insects' guts. The researchers confirmed the specificity of these associations between splice forms and particular pathogens by selectively silencing the exon transcripts induced by different bacteria. Disabling bacteria-specific exons reduced binding for the target bacteria but had no effect on other bacteria.

Altogether, these results show that infection-induced *AgDscam* splicing creates receptors better equipped to recognize—and defend against—the invading pathogen. Cells generated different splice-form repertoires depending on the source of infection. Alternative splicing allows the insect to vastly increase its repertoire of pattern recognition receptors from one single gene and thereby fight infection more efficiently. This work suggests that a better understanding of how *A. gambiae*'s hypervariable receptor AgDscam recognizes the *Plasmodium* parasite might suggest novel ways to control malaria by targeting the parasite inside its mosquito host.

## 

**Figure:**
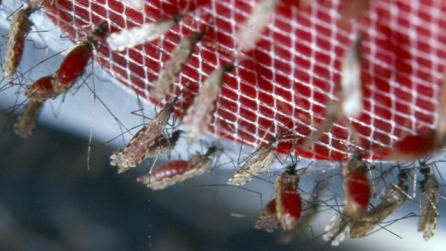
Blood-sucking Anopheles gambiae mosquitoes possess a hypervariable pattern recognition receptor capable of producing thousands of possible alternative splice forms to fight infection. (Photo: Johns Hopkins Malaria Research Institute)

